# Lamin B is a target for selective nuclear PQC by BAG3: implication for nuclear envelopathies

**DOI:** 10.1038/s41419-018-1255-9

**Published:** 2019-01-10

**Authors:** Manish K. Gupta, Jennifer Gordon, Gregory M. Glauser, Valerie D. Myers, Arthur M. Feldman, Joseph Y. Cheung, Kamel Khalili

**Affiliations:** 1 Department of Neuroscience, Center for Neurovirology, Katz School of Medicine at Temple University, Philadelphia, PA 19140 USA; 2 Department of Medicine, Katz School of Medicine at Temple University, Philadelphia, PA 19140 USA; 3 Center for Translational Medicine, Katz School of Medicine at Temple University, Philadelphia, PA 19140 USA

## Abstract

Nuclear envelopathies are recognized genetic disorders affecting individuals with mutations in their genes encoding members of the lamin family of nuclear envelope proteins that are responsible for maintaining the architectural structure of the nucleus. Irregularity in shape and size of the nuclei, nuclear membrane rupture, and appearance of micronuclei in the cytoplasm are among the pathological features of the syndrome. Here, we demonstrate that Bcl2-associated anthanogene-3 (BAG3), a stress-induced co-chaperone protein that by association with heat-shock protein 70 (HSP70) participates in regulation of autophagy, plays a critical role in the integrity of the nuclear membrane in cardiomyocytes. Cells subjected to proteotoxic stress or BAG3 downregulation show perinuclear accumulation of the aberrant ubiquitinated proteins that are often associated with the appearance of misshapen, enlarged, and elongated nuclei. There were dense accumulations of lamin B in the perinuclear area and distribution of lamin B-positive micronuclei in the cytoplasmic space, indicative of nuclear envelope rupture. Overexpression of BAG3 in cells under proteotoxic stress ameliorated pathological nuclear morphology and reduced cytoplasmic distribution of the micronuclei particles. Subcellular co-localization and co-immunoprecipitation demonstrated interaction of lamin B with the BAG domain of BAG3 and HSP70, suggesting the importance of BAG3 in the selective clearance of a surplus of aggregated lamin B that is generated during stress conditions. Our findings define a novel role for BAG3 in nuclear protein quality control and suggest an alternative pathogenetic pathway that contributes to the development of nuclear envelopathies.

## Introduction

The integrity of the nuclear membrane is critically important for separating nuclear function from cytoplasmic function. The inner surface of the nuclear membrane, by providing a docking site for chromatin, influences many nuclear functions such as epigenetic regulation of transcription, DNA replication, genome stability, and DNA repair among others^1–3^. The list of the nuclear membrane-associated diseases, termed nuclear envelopathy syndrome, has greatly increased in recent years^4,5^. Mutations in genes encoding type V intermediate filaments, including lamin A/C proteins, that form a meshwork positioned under the nuclear inner membrane, called lamina, have been associated with several rare heart-associated fatal progeria diseases, also known as laminopathy^4,6–8^. Common features consist of aberrancy in the structure and function of nuclear proteins caused by the mutation that impacts the integrity of the nuclear membrane, hence compromising nuclear-cytoplasmic communications, intra-nuclear homeostasis, and chromatin stability^7,9–14^. On the other hand, lamin B that is not associated with any genetic diseases that influence the heart is critically important in the processes that regulate correct assembly of nuclear envelopes, DNA replication, and cell survival. Several studies suggested a role for lamin B in cell senescence that is liked to chromatin reorganization, changes in gene expression, and aging^15–17^. Mutations in lamin B have been associated with neural tube defects, effects on myelin sheaths, and lipodystrophies^18^.

Bcl2-associated anthanogene-3 (BAG3) is a 575-amino-acid protein that is expressed predominantly in the heart and skeletal muscle, and in many cancers. A member of the BAG family of proteins, BAG3 has received recent attention in the heart due to his importance in autophagy and protein quality control (PQC)^19,20^ PQC is comprised of molecular chaperones and intracellular proteases in the cytosol and endoplasmic reticulum^21^. While one responsibility of chaperones relates to their assistance in folding of nascent proteins, their primary function is concentrated, together with co-chaperones and proteases, on the protection of cells against misfolded and excess damaged proteins caused by the internal and external stressor^22,23^. BAG3 is a stress-induced protein that interacts with HSP70 (heat-shock protein 70) through its highly conserved BAG domain, positioned in the C terminus of the protein, and regulates the function of the HSP70 molecular chaperone in clearance of damaged proteins and maintenance of protein homeostasis through the autophagy mechanism^24,25^. Earlier studies have linked heterozygous mutations in BAG3 to dilated cardiomyopathy^25,26^. Homozygous ablation of the BAG3 gene in a mouse model caused a broad range of pathology in muscle fiber, manifested as sarcomere disarray, apoptotic nuclei, cardiac dysfunction, and premature death^27^. Homozygosity of the human E455K mutation in mice disrupted the interaction between BAG3 and HSP70, decreased levels of small HSPs (sHSPs), and adversely affected autophagy, suggesting that interaction between BAG3 and HSP70 is essential for BAG3 to stabilize sHSPs^28^. Recent studies have demonstrated that haploinsufficiency of BAG3 was associated with diminished left ventricular function by 10–12 weeks of age that was accompanied by diminished autophagy and increased apoptosis but without evidence of myofibrillar dissary^29^.

More recently, we demonstrated that levels of BAG3 were reduced in mice with diminished cardiac function secondary to a myocardial infarction and that restitution of normal levels of BAG3 using an adeno-associated virus restored near-normal left ventricular function^30^. Further, we found that under stress conditions caused by hypoxia/re-oxygenation, BAG3 translocates to the nucleus, suggesting a protective role for BAG3 in maintaining normal nuclear function by mechanism that remains unexplored^31^. While the functional importance of BAG3 in the nucleus remains to be determined, one may envision a protective role for BAG3 in the maintenance of nuclear homeostasis by controlling PQC and nuclear autophagy. To address this question, we focused our attention on lamin B and found that changes in the environment that altered the clearance of misfolded lamin B might have a distinct phenotype such as misshapen nuclei. To assess the involvement of BAG3 in this event, we took two approaches: one was to alter the cellular content of BAG3 and thereby alter stress-induced autophagy and the other alternatively inhibit proteosomal PQC by inhibiting the proteasome with MG125. Here, we demonstrate that downregulation of BAG3 and/or inhibition of proteasomal degradation pathway result in excess perinuclear presence of lamin B, enlarged and misshaped nuclei, and nuclear envelope rupture. These observations suggest that in cells in which the proteasomal degradation pathway is inhibited, BAG3 overexpression ameliorated the pathological nuclear enlargement and minimized nuclear envelope rupture. Mechanistically, we demonstrated that lamin B co-localized and associated with the BAG domain of BAG3 together with the molecular chaperone HSP70. These results suggest a novel function for BAG3 in maintaining the level and quality of lamin B, thereby promoting the integrity of the nuclear envelope under stressful conditions.

## Results

**Fig. 1:**
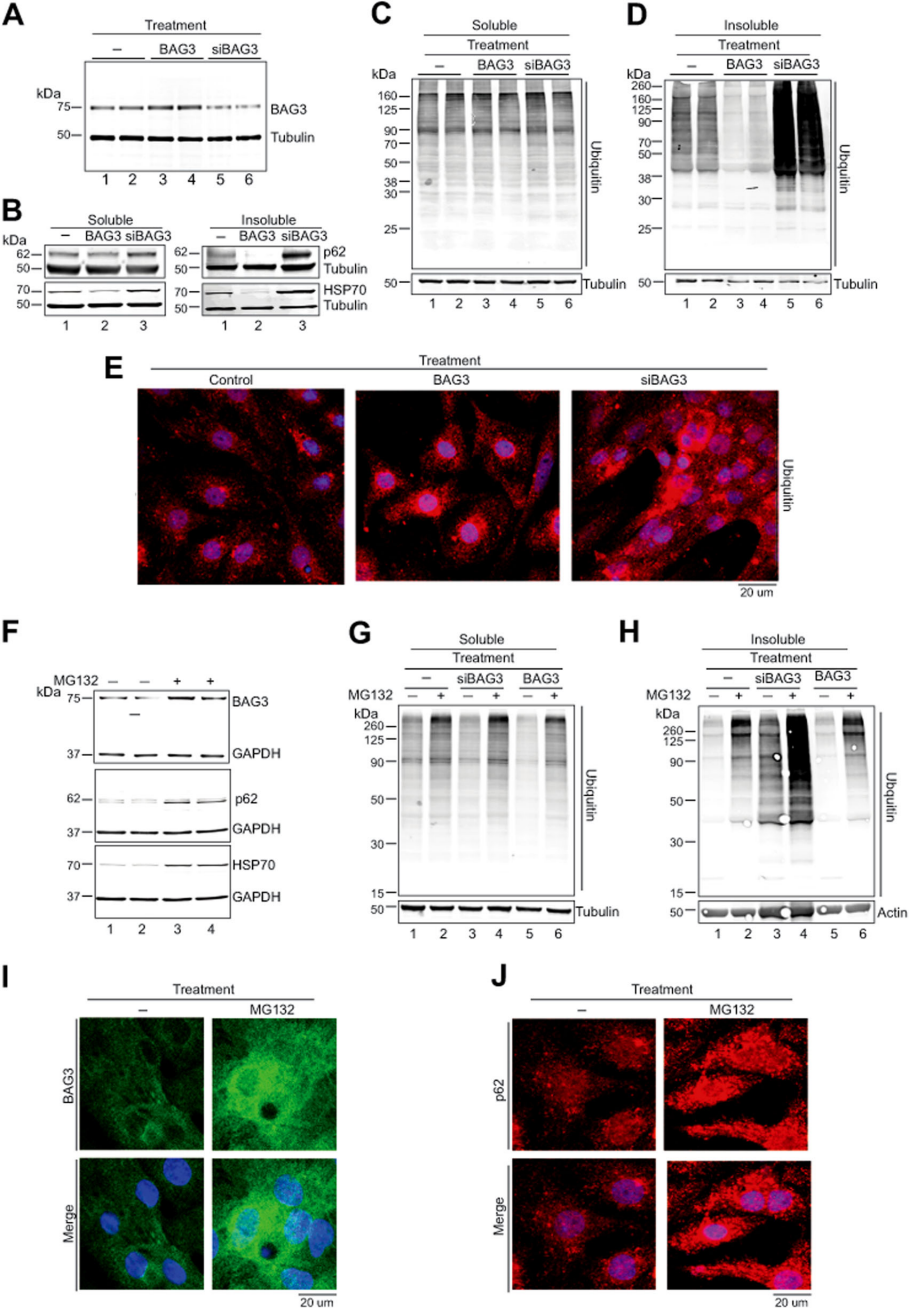
BAG3 is important for maintaining cellular protein quality control. **a** Reduced expression of BAG3 disrupts cellular proteostasis and causes accumulation of aggregated proteins. Cardiomyocytes were transduced with Ad-GFP, Ad-BAG3, and Ad-siBAG3 for 48 h. Expression of BAG3 was determined in the total cellular fraction. **b–d** Soluble and insoluble fractions were prepared from the transfected cardiomyocytes and expression of p62, HSP70, and ubiquitin was determined by western blotting. Expression was normalized with Tubulin. **e** Representative images show that knockdown of BAG3 causes accumulation of ubiquitin-positive cellular aggregates in cardiomyocytes. Cardiomyocytes were transduced with the respective adenovirus and cells were fixed with 4% PFA. Fixed cells were subsequently stained with ubiquitin antibody (red) and nuclei were stained with DAPI. **f–h** Inhibition of proteasomal function upregulates BAG3 expression in the cardiomyocytes. Cardiomyocytes were treated with MG132 (20 μM) for 6 h and expression of BAG3 was evaluated by western blot. Expression of p62, HSP70, and ubiquitin was assessed by western blot in cardiomyocytes treated with MG132 for 6 h. **g–h** Knockdown of BAG3 increases accumulation of ubiquitinated proteins in the insoluble fraction. NRVCs were transduced with adenovirus for 48 h and treated with MG132 before protein isolation. Western blot was done with ubiquitin antibody in the soluble and insoluble fractions. **i** Representative microscopy images show that inhibition of proteasomal function causes upregulation of BAG3 protein in cardiomyocytes. Cardiomyocytes were treated with MG132 for 6 h and cells were fixed with 4% PFA. Fixed cells were then stained with BAG3 antibody (green) and nuclei were stained with DAPI (blue). **j** Fixed cells were stained with p62 antibody (red) and nuclei were stained with DAPI

We evaluated the potential role of BAG3 in lamin B homeostasis of neonatal rat ventricular cardiomyocytes (NRVCs) by manipulating its intracellular levels with Ad-siBAG3 or Ad-BAG3 (Fig. 1a) and assessing accumulation of protein aggregates. Changes in the level of BAG3 correlated inversely with the levels of p62 and HSP70, known regulators of autophagy and PQC pathway, in the insoluble protein fractions (Fig. 1b). In addition, knockdown of BAG3 caused accumulation of ubiquitinated proteins in the insoluble fraction, whereas overexpression of BAG3 reduced the abundance of the ubiquitinated proteins in the insoluble fraction, verifying regulation of PQC by BAG3 in cardiomyocytes (Fig. 1c, d). Quantitative analyses of these results are presented in Supplementary Fig. 1. In BAG3-knockdown NRVCs, ubiquitinated protein aggregates accumulated in the perinuclear space (Fig. 1e). Inhibition of proteasomal degradation pathway by the proteasome inhibitor MG132 resulted in the increased levels of BAG3, HSP70, and p62 (Fig. 1f), as well as enhanced accumulation of ubiquitinated proteins in cardiomyocytes (Fig. 1g, h). Under normal conditions, the control cells exhibited lower levels of the ubiquitinated proteins in insoluble fractions than those in the soluble fractions, pointing to the efficient clearance of the aggregated proteins (Fig. 1, compare lane1 in panels g and h). Inhibition of proteasome degradation pathway resulted in more accumulation of the ubiquitinated proteins in the insoluble fractions (Fig. 1, compare lane 2 in panels g and h), suggesting that despite higher levels of BAG3 and p62 (Fig. 1f) in the cytoplasm and nuclear space (Fig. 1i, j), PQC is compromised in cells with the impaired proteasome pathway.

**Fig. 2:**
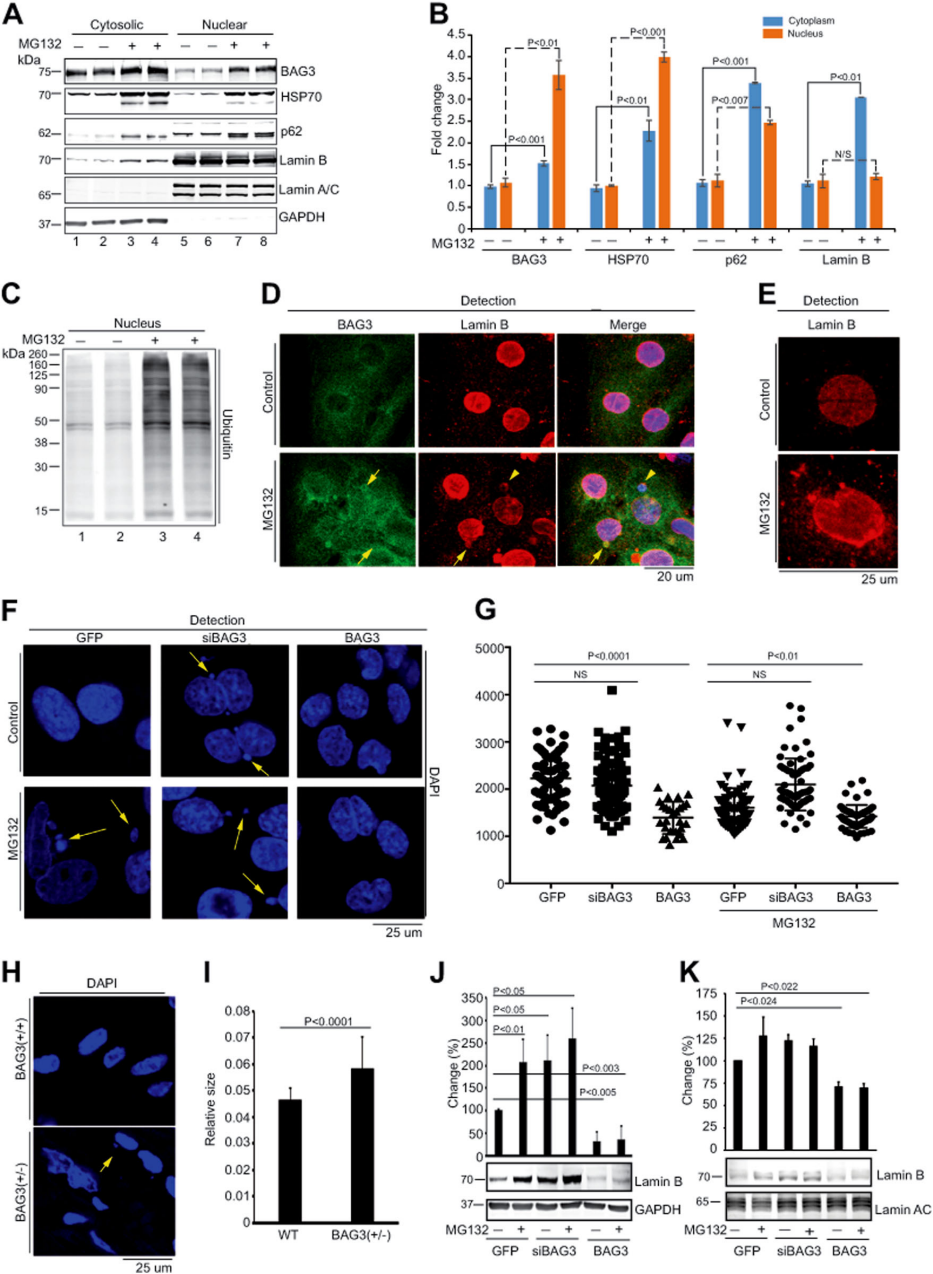
BAG3 is important for removal of nuclear protein during proteotoxic stress. **a**, **b** Western blots show that due to proteasomal inhibition, expression of BAG3 and lamin B was upregulated in the cytosolic fraction as well as in the nuclear fraction. NRVCs were treated with the proteasomal inhibitor MG132 (20 µM) for 6 h and cells were fractionated into cytosolic and nuclear fractions. Expression of BAG3 and lamin B, HSP70, and p62 was determined by western blot in the cytosolic as well as in nuclear fraction by BAG3, lamin B, HSP70, and p62 antibodies, respectively. Expression of cytosolic and nuclear proteins was normalized with GAPDH and lamin A/C expression, respectively. Graph shows the quantification of BAG3, HSP70, p62, and lamin B expression in the cytosol as well as in the nuclear fraction. **c**, **d**) Proteasomal inhibition results in the accumulation of ubiquitinated proteins in the nuclear fraction. Cardiomyocytes were treated with MG132 (20 µM) for 6 h, and cytosolic and nuclear fractions were prepared. Western blot was done with the ubiquitin antibody. **d** Representative images show that inhibition of proteasomal function caused accumulation of micronuclei in the cytosol. Cardiomyocytes were treated with the proteasomal inhibitor for 6 h and fixed with 4% PFA. Fixed cells were stained with the BAG3, lamin B, and nuclei were stained with DAPI. **e** Representative images show that inhibition of proteasomal function with MG132 causes dysregulation of nuclear shape. MG132-treated cardiomyocytes were fixed with 4% PFA and stained with the lamin B (red). **f** Representative images show that knockdown of BAG3 or the treatment of the cells with MG132 affects the size and shape of the nuclei and causes accumulation of micronuclei in the cytoplasm. Cardiomyocytes were transduced with the respective adenoviral construct for 48 h and treated with MG132 (20 µM) for 6 h. Cells were fixed with 4% PFA and stained with DAPI. **g** Quantification of the results (shown in panel f) depicting nuclear area of the control and treated cells (*n* = 50–100 in each group) as measured by the ImageJ software. **h** Representative images show the nuclear morphology in wild-type and in BAG3 (+/−) mice heart. **i** Quantitative presentation of the results shown in panel h. **j** Examination of lamin B in the cytosolic fractions of NRVCs under the conditions that the level of BAG3 is changed and the cells are under the stress upon the treatment with MG132. Cardiomyocytes were transduced with the respective adenovirus for 48 h and cells were treated with MG132 (20 µM) for 6 h. **k** Expression of lamin B (under similar conditions as described in **j**) in nuclear-insoluble fractions. The level of lamin A/C, which was shown to remain unchanged, served as an internal control. NS not significant

Next, we investigated the subcellular levels of BAG3, HSP70, and several nuclear membrane proteins (lamin A/C and B) in MG132-treated NRVCs. The level of BAG3 expression is increased in both cytosolic and in nuclear fractions of cells under proteotoxic stress (Fig. 2a, b). A coordinated enrichment in BAG3, HSP70, and p62 in the nuclear fraction of MG132-treated NRVCs suggested that PQC was activated in the nuclei of the treated cells. In addition, the nuclear envelope protein, lamin B, but not lamin A/C, was increased in the cytosolic fraction of the MG132-treated NRVCs (Fig. 2a, b). Furthermore, the level of ubiquitinated proteins was found to be increased in the nuclear fractions of the MG132-treated cells, but not in the MG132-untreated cells (Fig. 2c).

Microscopic imaging studies revealed the nuclear presence of BAG3 and the appearance of BAG3 containing puncta as well as small cytoplasmic lamin B-positive particles of various sizes that were stained with 4′, 6-diamino-2-phenyl indole (DAPI) in MG132-treated cells (Fig. 2d). Appearance of DAPI-positive particles, called micronuclei, in the cytoplasmic space of the MG132-treated cells indicated nuclear membrane rupture resulting in scattering of nuclear materials in the cytoplasm. In addition, MG132-treated NRVCs harboring enlarged and misshaped nuclei with dense perinuclear accumulation of lamin B were occasionally observed (Fig. 2e); some of the misshaped nuclei were attached to or in juxtaposition of micronuclei (Fig. 2f). Misshaped and slightly enlarged nuclei, with and without micronuclei attachment, were also observed in NRVCs in which BAG3 was downregulated with siBAG3 RNAs (Fig. 2f, g). Conversely, BAG3 overexpression noticeably diminished the appearance of micronuclei and preserved normal size of the nuclei (Fig. 2f, g). In agreement with the results in NVRCs, in adult cardiac ventricular cells isolated from BAG3+/− mice, deformed and elongated nuclei distinct from those seen in the control age-matched cells were observed (Fig. 2h, i).

Using a fundamentally different approach to demonstrate the importance of BAG3 in nuclear homeostasis, the DNA sequence corresponding to the BAG3 domain of the BAG3 gene was deleted in a mouse muscle cell line, C2C12, using the CRISPR/Cas9 gene-editing technique (Supplementary Fig. 3). Consistently enlarged and misshaped nuclei in cells with no BAG3 expression were observed (Supplementary Fig. 4).

Cytosolic accumulation of lamin B was observed in NRVCs in which the proteasomal degradation pathway was inhibited or BAG3 was downregulated (Fig. 2j). On the other hand, overexpression of BAG3 significantly reduced cytosolic accumulation of lamin B in MG132-treated NRVSc (Fig. 2j). Under similar conditions, the level of lamin B in insoluble nuclear fraction was slightly increased upon the treatment with MG132 (50%) or cells treated with siBAG3 (35%) (Fig. 2k). Overexpression of BAG3 caused a modest decrease (20%) in the level of lamin B detected in the insoluble nuclear fraction (Fig. 2k). Taken altogether, these observations provide evidence that BAG3 is critically important for the maintenance of nuclei homeostasis during the proteotoxic stress.

**Fig. 3:**
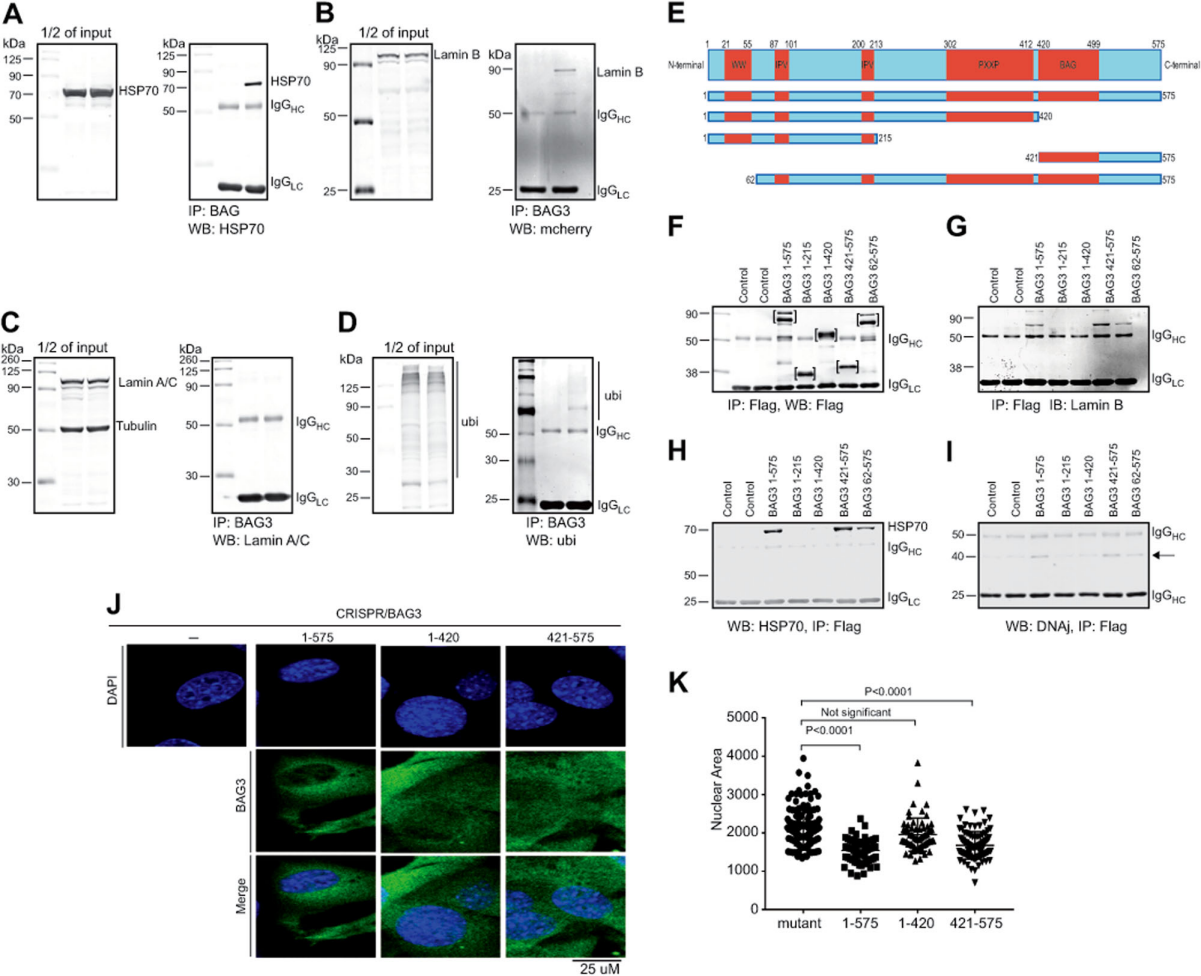
BAG3 interacts with nuclear protein lamin B using BAG domain. **a** Co-immunoprecipitation studies show that HSP70 co-immunoprecipitated with BAG3. HEK293 cells were transfected with BAG3 plasmid for 48 h and immunoprecipitation was done with FLAG-tag antibody. Western blot was done with HSP70 antibody. **b**, **c** Immunoprecipitation study shows that BAG3 can interact with nuclear envelop protein lamin B but not with lamin A/C. HEK293 cells were co-transfected with BAG3 and lamin B-mCherry or BAG3 and lamin A/C-mCherry. Immunoprecipitation was done with the FLAG antibody and western blots were done using lamin B or lamin A/C antibody, respectively. **d** Western blot shows that BAG3 interacts with the ubiquitin. HEK293 cells were co-transfected with BAG3 and ubiquitin expressor plasmids. Immunoprecipitation was done with FLAG antibody and western blots were done with ubiquitin antibody. **e** Schematic diagram shows different domains of BAG3. **f** Western blot analysis showing detection of full-length BAG3 and the BAG3 deletion mutants by anti-FLAG. **g–i** Co-immunoprecipitation of BAG3 (using anti-FLAG antibody) followed by western blot for detection of lamin B as well as the BAG3 partners, HSP70 and DNAJ, respectively. **j** Representative images show that BAG domain can restore the wild-type phenotype in the CRISPR-mediated BAG3 mutant C2C12 cells. Wild-type and mutant C2C12 cells were transfected with the mutant and wild-type BAG3 gene using Lipofectamine for 48 h. Cells were fixed with 4% PFA and immunocytochemistry was done with the FLAG-tag antibody (green) and nuclei were stained with DAPI (blue). Images were captured with confocal microscopy. **k** Quantification of the results (shown in **j**) in which nuclear area was measured using the ImageJ software (*n* = 100 cells, in each group)

The subcellular co-localization of BAG3 with lamin B suggested that lamin B may physically associate with BAG3 and/or HSP70, both of which are important for the clearance by PQC. Results from co-immunoprecipitation studies with HEK293 cells expressing BAG3 with and without lamin B1–10 showed the association of BAG3 with HSP70 and lamin B but not lamin A/C (Fig. 3a–c). We also found interaction of BAG3 with ubiquitinated proteins in these cells (Fig. 3d). The BAG3 molecule has many domains that allows its interaction with several important regulatory proteins and participation in apoptosis and autophagy pathways^26,32,33^. To identify the domain within BAG3 that associates with lamin B, we expressed a series of FLAG-tagged BAG3 mutants (Fig. 3e) along with the mCherry-lamin B construct in HEK293 cells (Fig. 3f). Results of the co-immunoprecipitation assay showed that lamin B specifically recognizes a region proximal to the C terminus of BAG3 that overlaps the BAG domain (Fig. 3g). As expected, HSP70 as well as DNAj associated with the BAG domain of BAG3 (Fig. 3h, I). Expression of the full-length (1–575) and a truncated BAG3 protein encompassing the BAG domain (421–575), but not a mutant BAG3 with no BAG domain (1–420), improved the size of the nuclei in the C2C12 cells whose BAG3 expression was ablated by CRISPR editing (Fig. 3j, k).

## Discussion

Under stress conditions, cardiomyocytes accumulate aggregates of damaged or misfolded proteins that become toxic to the cells and often promote the cell death pathway that in turn can trigger cardiomyopathy and heart failure^34–37^. In balance, cells have evolved compensatory cell survival mechanisms such as protein control pathway, PQC, that involve activation of chaperone and co-chaperone networks of inducible proteins. These molecules, by assisting proper folding of nascent proteins, refolding misfolded proteins, and removing the accumulated protein aggregates from the cytoplasm, ensure healthy intracellular homeostasis^23,38^. In this study, we show that during the proteotoxic stress induced by MG132, cells trigger the compensatory pathway that includes increased expression and nuclear presence of co-chaperone BAG3 and its partner, HSP70, along with autophagy regulator, p62, to remove damaged, overexpressed, and dysfunctional proteins from the nuclear environment. While the mechanism that results in induction of BAG3 by MG132 remains to be fully determined, one may envision that, consistent with earlier observations in HeLa cells^39^, treatment with MG132 induces BAG3 expression at the transcription level, in part through activation of HSF1. In support of this notion, our preliminary data show induction of BAG3 RNA levels in rat cardiomyocytes after treatment with MG132 (unpublished data). In fact, the perinuclear appearance of the ubiquitinated proteins that are programmed for degradation by the proteosomal pathway, yet aborted by the proteasome inhibitor, is indicative of activation of the PQC pathway in cells under proteotoxic stress. Detection of ubiquitinated proteins in the nuclear fractions of cardiomyocytes, when BAG3 becomes silent, is suggestive of its involvement in clearance of nuclear proteins by the PQC pathway.

One major unexpected finding from our study is the appearance of irregular and enlarged nuclei, perinuclear accumulation of lamin B, and the appearance of micronuclei containing lamin B in the cytoplasm of cells in which either BAG3 level was reduced and/or the protein degradation pathway was pharmacologically interrupted. Interestingly, these pathological changes were ameliorated by the BAG3 overexpression, thereby underscoring the importance of BAG3 in nuclear homeostasis. Perhaps, it is noteworthy to mention that NRVCs are a primary non-replicating semi-differentiated cardiomyocyte cells that have many characteristics of cardiac cells including the excitation–contraction coupling. At present, the impact of BAG3 on lamin B in the other differentiated cells and/or rapidly replicating cells remains unknown. Having said that, our observations in the replicating mouse-derived myoblast C2C12 cell line revealed a modest improvement in the size of the nucleus of CRISPR-mediated BAG3-null cells upon expression of BAG3 in these cells (Supplementary Figs. 3 and 4).

The nuclear envelope has received much attention in recent years due to the association of defects in nuclear proteins with several fatal human diseases^5^. The nuclear envelope is known for providing mechanical support to nuclei and, through nuclear lamina meshwork underlying the inner nuclear membrane, communicates with a variety of nuclear functions including DNA repair, chromatin structure, telomere maintenance, cell cycle progression, and others^1,2^. Laminopathies are a group of more than 11 tissue-specific degenerative diseases linked to inherited mutations in lamin A^4,6,8^. In Hutchinson–Gilford Progeria syndrome (HGPS), the premature aging syndrome, a single mutation at position 608, results in an aberrant splicing and deletion of 50- amino-acid residues from the C terminus of the protein keeping the protein in the farnesylated form^6,40^. While no specific mutation in the coding sequence of lamin B has been linked to human cardiac diseases, increased expression of lamin B has been reported in peripheral leukocyte as well as in a variant of autosomal dominant leukodystrophy^18^. Overexpression of lamin B has been observed in ataxia telangiectasis^41^ and that correcting its levels appears to mitigate nuclear misshaping and premature senescence of patients’ cells^42,43^. These observations suggest that increased expression of lamin B may be detrimental to cells and perturbs the homeostasis of nuclear function as suggested by its irregular shape and size.

Our current results delineate a novel and heretofore not described major function of BAG3 which is to maintain the quality and levels of functional nuclear lamin B. In cells under proteotoxic stress, especially when BAG3 levels are reduced, perinuclear accumulation of lamin B promotes enlarged nuclear morphology and nuclear envelope rupture, thereby exposing chromosomal DNA to the cytoplasmic environment (micronuclei) and further DNA damage. Overexpression of BAG3 ameliorates the pathological changes including normalization of nuclear sizes and minimizing nuclear envelope rupture (as judged by the reduction in micronuclei). Collectively, our results suggest that an additional process beyond the mutations in nuclear lamins may participate in the pathophysiology of nuclear envelopathies. In fact, earlier studies demonstrated that aberrant expression of Sun1 is a critical pathogenic event important for the development of HGPS^44^. In light of these observations, one may speculate a potential role for BAG3 in cellular senescence and its use for improving longevity in human.

## Materials and methods

### Cell culture and treatment

Cardiomyocytes were isolated from rat ventricular tissue of 1–2-day-old Harlan Sprague–Dawley rats by collagenase and trypsin digestion as described earlier^45^. Isolated primary NRVCs were plated at a density of 1.5 × 10^6^ cells in 10 cm plates, or 1.5 × 10^5^ cells in two-well chamber slides. Cells were grown initially in minimum essential medium-α (Gibco) plus 10% fetal bovine serum (FBS) for 24 h, after which the medium was replaced with high glucose Dulbecco's modified Eagle's medium (DMEM) (Gibco) plus 2% FBS and supplemented with the 50 μg/ml gentamicin (Gibco). Other cell lines such as HEK293 (ATCC) or C2C12 (ATCC) were grown in DMEM supplemented with 10% FBS and 50 μg/ml gentamicin (Gibco). Cells were treated with proteasomal inhibitor 20 µM MG132^45^ (Nalgene) for 6 h as needed and control cells were treated with dimethyl sulfoxide.

### Generation of cardiac-specific BAG3-haploinsufficient mice

Animal handling and care is in accordance with the National Institute of Health Guide for the Care and Use of Laboratory Animals. All animal procedures were approved by the Institutional Animal Care and Use Committee at Temple University. BAG3-haploinsufficient mice were generated by crossing the BAG3 single allele-floxed (BAG3^fl/+^) mice with αMHC-Cre mice as described earlier^29^. All BAG3-knockout (−/−) and BAG3-haploinsufficient (+/−) and wild-type (WT) littermates were maintained in C57BL/6 background. Mice were genotyped by PCR as described earlier^29^.

### Knockdown and overexpression of BAG3 in NRVCs

BAG3 protein was knocked down by recombinant adenovirus containing small specific interfering RNA (Ad-siBAG3)^46,47^. BAG3 protein was also overexpressed by adenovirus (Ad-BAG3) that encodes human WT BAG3 via the cytomegalovirus (CMV) promoter^46^. At 24 h after plating, NRVCs were transduced with adenovirus. For transduction, primary cells were incubated with adenovirus for 2 h in DMEM without serum. Transduction medium was replaced with DMEM supplemented with 2% FBS and 50 µg/ml gentamicin (Gibco). Control cells were transduced with adeno-null (Ad-null) or adeno-GFP virus (Ad-GFP). For overexpression in cell lines, plasmids were transfected with Lipofectamine 2000 (Life Technologies).

### Protein isolation and western blotting

Western blot was performed as described earlier with some modification. Cultured cells were washed two times with 1× phosphate-buffered saline (PBS) and lysed in RIPA buffer (150 mM NaCl, 1.0% IGEPAL® CA-630, 0.5% sodium deoxycholate, 0.1% sodium dodecyl sulfate (SDS), 50 mM Tris, pH 8.0) containing 1× mammalian protease inhibitor (Sigma). Unlysed cells and debris were removed from the total protein lysate by centrifugation at 10,000 × *g* for 10 min at 4 °C. Isolated supernatant proteins were subjected to SDS-polyacrylamide gel electrophoresis (SDS-PAGE) and blotted to nitrocellulose membrane (LI-COR). After transfer, membrane was blocked with 1× blocking buffer (LI-COR) in 1× PBS at room temperature. Membranes were incubated with primary antibody diluted in 1× blocking buffer overnight. Membranes were washed with 1× PBST (Tris-buffered saline + Tween-20) for three times followed by incubation with a DyLight-conjugated secondary antibody (Invitrogen) at room temperature for 2 h. The membrane was washed three times with 1× PBST, one time with 1× PBS and scanned on Odyssey CLx scanner using the Image Studio software (LI-COR). Soluble and insoluble fractions of NRVCs were prepared as described earlier^45^. NRVCs were fractionated into cytosolic and nuclear fraction using NE-PER Nuclear and Cytoplasmic Extraction Kit (Thermo Fisher).

### Immunofluorescence microscopy

Immunofluorescence microscopy was performed as described earlier. In brief, cultured NRVCs or cell lines were washed two times with 1× PBS and cells were fixed with 4% paraformaldehyde (PFA) in PBS and incubated at room temperature for 10 min. Fixed cells were washed two times with 1× PBS and permeabilized with 0.5% Triton X-100 at room temperature for 10 min. After washing two times with 1× PBS, cells were incubated with 0.1 mol/l glycine buffer (pH 3.5) at room temperature for 30 min. Cells were washed two times with 1× PBS and blocked with blocking buffer (1% bovine serum albumin, 0.1% Tween-20 in 1× PBS) for 1 h at room temperature. Cells were incubated with primary antibodies in blocking buffer at 4 °C overnight. Cells were washed three times with 1× PBS and incubated with Alexa-Fluor-conjugated secondary antibody (Life Technologies) for 1 h at room temperature. Cells were washed three times with 1× PBS and slides were mounted with Vectashield Hard Set mounting medium (Vector Labs), and allowed to dry at room temperature for 30 min. In some experiments, cells were stained with multiple primary antibodies. In those case, cells were re-blocked after the first secondary antibody treatment with blocking buffer for 30 min at room temperature. Stained cells were analyzed under 710 confocal microscope (ZEISS). Images were analyzed with the ImageJ software (NIH).

### Immunohistochemistry

For histopathological examination, hearts were dissected out of mice and fixed in 10% buffered formalin. After fixation, hearts were divided in half, and remaining blood was removed. Fixed heart tissue was processed and blocked in paraffin for sectioning at 5 µm thickness. For staining, tissue sections were deparaffinized in xylene and rehydrated through decreasing-percent alcohols. Sections underwent antigen retrieval in heated citrate buffer, followed by blocking at room temperature for 1 h. Further, slides were stained with respective antibodies and nucleus was stained with DAPI as described earlier. Sections were mounted with coverslip using Vectashield Hard Set mounting medium (Vector Labs) and images were captured under 710 confocal microscope (ZEISS).

### Detection of apoptotic cells

Apoptotic cells were detected by TUNEL (terminal deoxynucleotidyl transferase dUTP nick-end labeling) assay. NRVCs were fixed with 4% PFA and apoptotic cells were detected by TUNEL Staining Kit according to the manufacturer's protocol. Cells were counter-stained by DAPI to detect nuclei. Images were captured under 710 confocal microscope (ZEISS) and analyzed by the ImageJ software (NIH).

### Plasmid construction

BAG3 deletion mutants were amplified by PCR from human BAG3 complementary DNA. Amplicons were cloned in the mammalian shuttle vector pShuttle-CMV (Agilent Technologies) downstream of the CMV promoter. For expression analysis of the mutant protein, a FLAG-tag (DYKDDDDK) was introduced at the N-terminal site. Expression of mutant protein was verified in HEK293 cells by Lipofectamine (Thermo Fisher) transfection. The plasmids mCherry-lamin B1-10 and mCherry-lamin A-C-18 were a kind gift from Michael Davidson lab (Addgene plasmid #55069), as were HA-p62 from Qing Zhong lab (Addgene plasmid #28027), and GFP-Ub from Nico Dantuma (Addgene #11928).

### Co-immunoprecipitation

HEK293 cells were transfected using Lipofectamine 2000 (Thermo Fisher). Forty-eight hours after transfection, cells were lysed in RIPA buffer (150 mM NaCl, 1.0% IGEPAL® CA-630, 0.5% sodium deoxycholate, 0.1% SDS, 50 mM Tris, pH 8.0) with 1× mammalian protease inhibitor. Lysates were cleared by centrifugation at 10,000 × *g* for 10 min at 4 °C. For interaction studies, proteins were precipitated from total protein lysate using respective antibodies tagged with magnetic beads (Sigma) according to the manufacturer's instructions. Immunoprecipitation was done in Tris (pH 7.5)-buffered saline with magnetic stand and bound proteins were eluted with 1× Laemmli buffer. Resulting immunoprecipitates were separated by SDS-PAGE and western blot was done with respective antibody to detect interaction.

### Generation of stable cells lines using CRISPR/Cas9

BAG3-mutated stable cell lines were generated using the CRISPR/Cas9 method in mouse muscle cell C2C12 (ATCC). To knockdown the BAG3 gene, two guide RNAs (gRNAs) were generated using the Benchling Software (Benchling). The following primers were used to generate gRNAs:

1. Bag3e4A/S/2—5′-AGAACCTGCAGCCCCCAAATC-3′.

2. Bag3e4A/S/2—5′-GAACCTGCAGCCCCCAAATCGG-3′.

3. Bag3e4B/S/2—5′-GATTCCGTAGACCCTGAAGG-3′.

4. Bag3e4B/S/2—5′-GGATTCCG TAGAC CCTGAAG-3′.

gRNAs were cloned in pX601-AAV-CMV::NLS-SaCas9-NLS-3xHA-bGHpA;U6::BsaI-sgRNA^48^ vector and transfected to C2C12 cells using Lipofectamine 2000 (Thermo Fisher). Plasmid pKLV-U6gRNA(BbsI)-PGKpuro2ABFP^49^ was also co-transfected with pX601-AAV-CMV::NLS-SaCas9-NLS-3xHA-bGHpA;U6::BsaI-sgRNA for puromycin-based (Gibco) selection. Cells were grown for another week and then puromycin (1 µg/ml) was added to select stable puromycin-resistant cells. Single-cell colonies were chosen and deletion of BAG3 gene was checked by PCR. BAG3 protein expression in mutant cells was verified by western blot using BAG3 antibody.

### Nuclear length and area analysis

To quantify the variation in nuclear length and area, C2C12 cells were grown in DMEM containing 10% FBS and cardiomyocytes were grown in DMEM containing 2% FBS. Cells were fixed with 4% PFA and stained with nuclear staining dye DAPI. Cells were mounted with Vectashield Hardset antifade mounting medium and imaging was done with confocal microscopy. Cardiomyocytes were also treated with adenovirus and proteasomal inhibitor MG132 as needed. Images were quantified with the ImageJ software and relative nuclear length (diameter) and relative nuclear area were measured in each group. For statistical analysis, we have measured 100 nuclei from each group.

### Statistical analysis

Analysis between the experimental groups was done by Student's *t* test on one-way ANOVA (analysis of variance) when multiple groups were compared. Values of *P* < 0.05 were considered significant.

## Supplementary information

Supplemental material and figures
