# Supplementary material for: Lamin B is a target for selective nuclear PQC by BAG3: implication for nuclear envelopathies
Source: Cell Death Dis. 2019 Jan 10;10(1):23. doi: 10.1038/s41419-018-1255-9 (PMC6328609; doi:10.1038/s41419-018-1255-9)
Supplement: Supplementary file 1 — Supplemental material and figures [file 41419_2018_1255_MOESM1_ESM.pdf]

July 20, 2018

Lamin B is a Target for Selective Nuclear PQC by BAG3: Implication for Nuclear Envelopathies

Running Title: Role of BAG3 in nuclear autophagy and lamin B quality control

SUPPLEMENTARY MATERIAL

Manish K. Gupta[1], Jennifer Gordon,[1] Gregory M. Glauser[1], Valerie D. Myers[2], Arthur M. Feldman[2], Joseph Y. Cheung,[3] Kamel Khalili[1†]

[1]Department of Neuroscience
Center for Neurovirology
Katz School of Medicine at Temple University
Philadelphia, Pennsylvania, 19140

[2]Department of Medicine,
Katz School of Medicine at Temple University
Philadelphia, Pennsylvania, 19140

[3]Center for Translational Medicine
Katz School of Medicine at Temple University
Philadelphia, Pennsylvania, 19140

†Corresponding author: Kamel Khalili, Ph.D.
Department of Neuroscience, Katz School of Medicine at Temple University, 3500 N. Broad Street, Philadelphia, PA 19140
Phone: 215.707.4500; Fax: 215.707.4888
Email: kamel.khalili@temple.edu

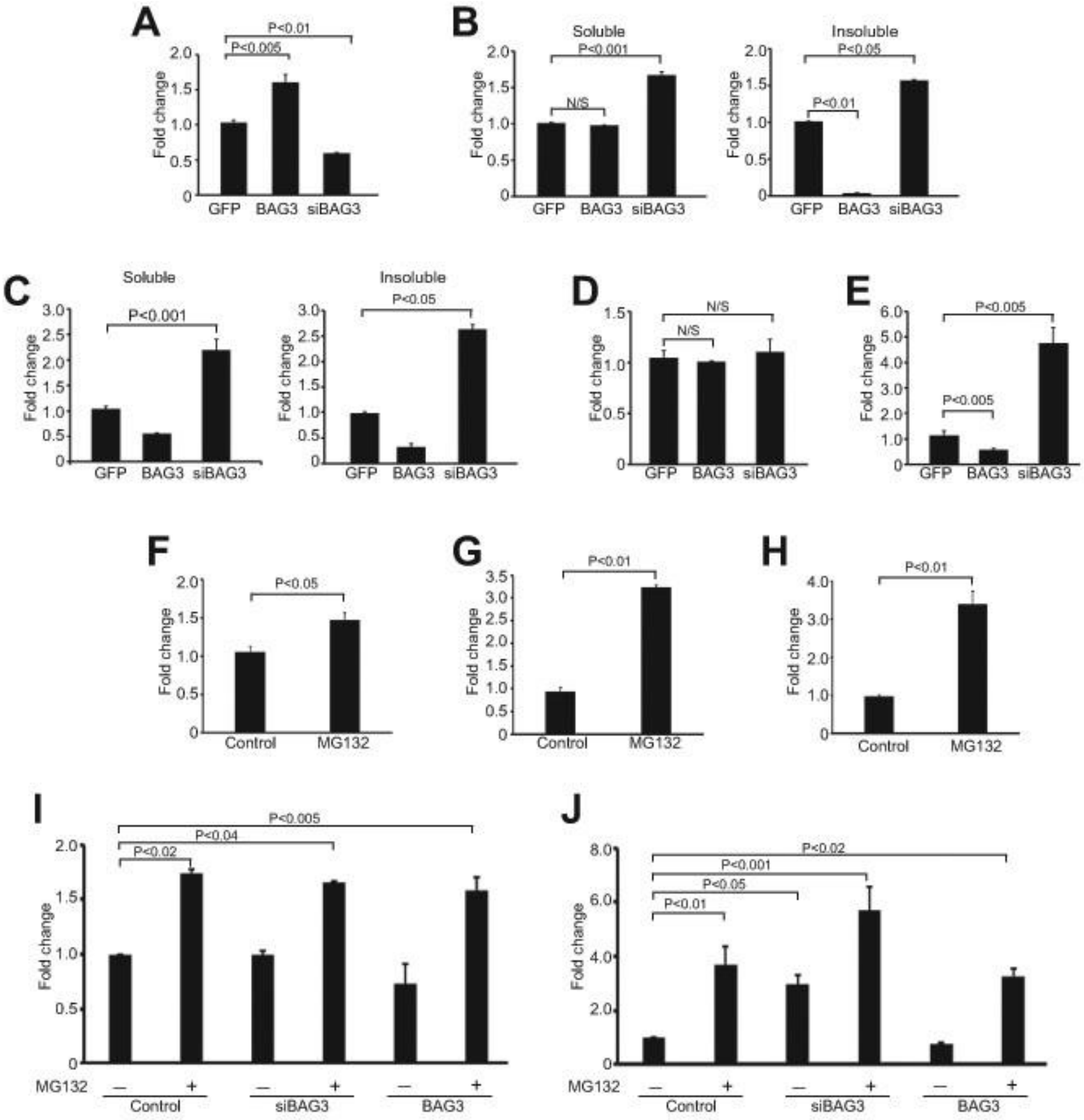

Supplemental Figure 1: Quantification of western blots in Figure 1: **(A).** Graph shows quantification of BAG3 expression Figure 1A. **(B)**. Graph shows quantification of p62 in the figure 1 B. **(C)**. Graph shows quantification of HSP70 expression in Figure 1B. **(D-E)**. Graph shows quantification of ubiquitin in the soluble and insoluble fraction of Figure 1 C-D. **(F-H).** Graphs show quantification of BAG3, p62 and HSP70 expression of figure 1F. **(I-J).** Graphs show the quantification of ubiquitin expression in figure 1G-H.

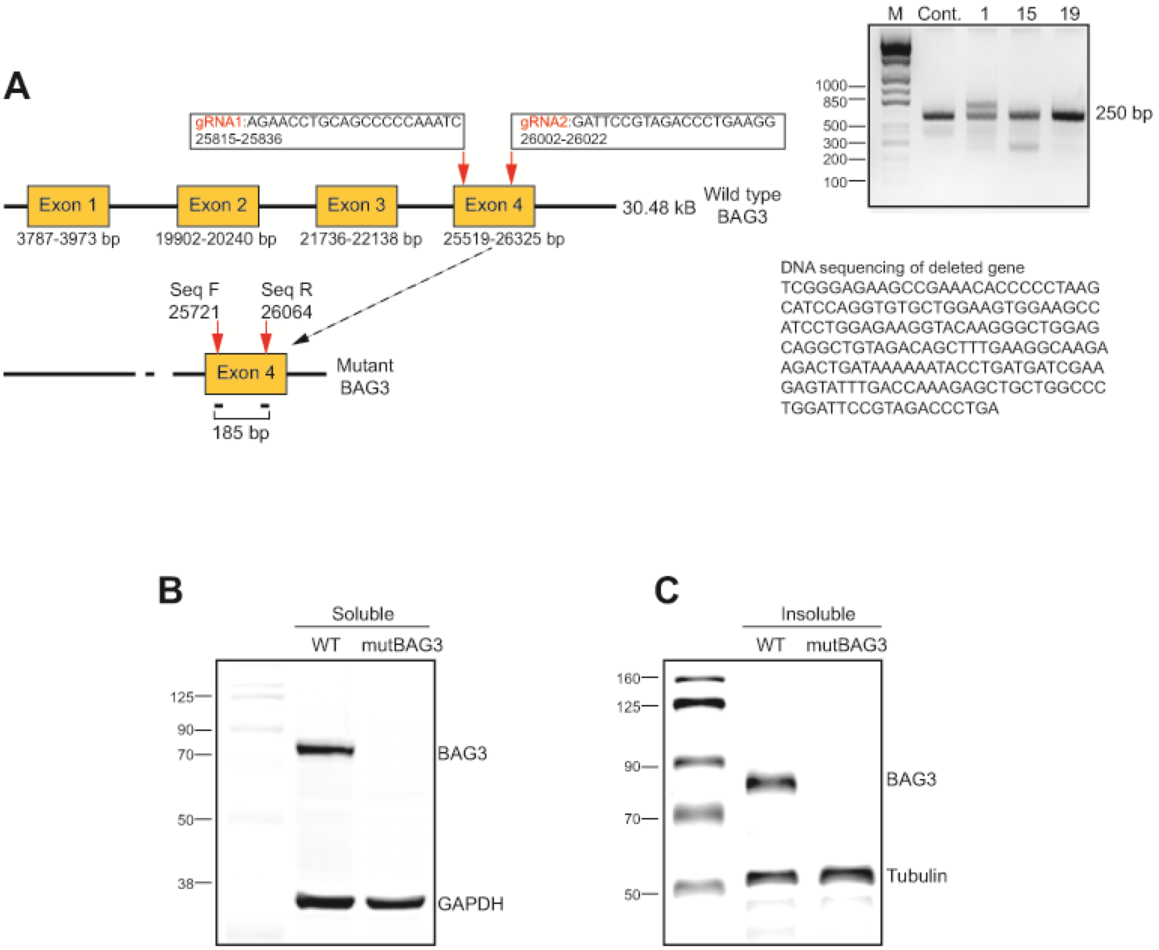

Supplemental Figure 2: Generation of BAG3 deleted stable cell lines. (A). Diagrammatic image shows the CRISPR/CAS9 targeted region of BAG3 gene. BAG3 mutant was generated in the C2C12 cell line using gRNAs for the Exon4 region. Deletion of BAG3 gene was confirmed by PCR amplification of mutant gene and Sanger DNA sequencing. **(B-C).** Expression of BAG3 gene was checked in the WT and BAG3 mutated C2-C12 cells by western blotting using BAG3 antibody.

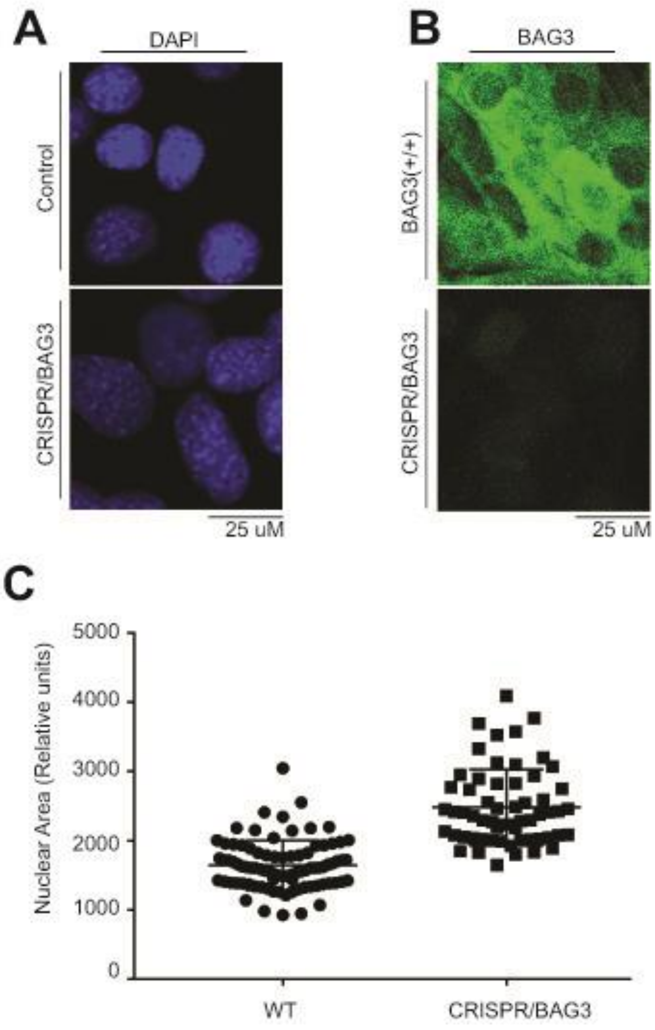

Supplemental Figure 3: Stable cell lines with BAG3 knockout were generated by CRISPR/CAS9 method as shown in Suppl. Fig 3. **(A).** Representative images show nuclear morphology in C2C12 cells. Nucleus of the fixed cells were stained with DAPI. **(B).** Expression of BAG3 in C2C12 WT and CRISPR mediated BAG3 gene knock out cells **(C).** Graph shows quantification of nuclear size in the C2C12 WT and BAG3 mutated cells
